# Modeling and Specification of Bootstrapping and Registration Design Patterns for IoT Applications

**DOI:** 10.1007/978-3-030-51517-1_5

**Published:** 2020-05-31

**Authors:** Mohamed Hadj Kacem, Imen Tounsi, Najeh Khalfi

**Affiliations:** 8 Digital Research Centre of Sfax, Sfax, Tunisia; 9 Institut Mines-Télécom, CNRS, Paris, France; 10 Université de Sherbrooke, Sherbrooke, QC Canada; 11 Digital Research Centre of Sfax, Sfax, Tunisia; 12 University of Sfax, Sfax, Tunisia; ReDCAD laboratory, University of Sfax, Sfax, Tunisia

**Keywords:** Design patterns, UML modeling, Event-B method, Pattern modeling, Formal specification

## Abstract

The architectures of software systems are becoming more complex, large, and dynamic. The design of these architectures allows architects to master building complex software systems. But, their informal description, may give rise to ambiguity, their understanding becomes more and more difficult and leads to the incorrect implementation of these software systems. There are many solutions allowing software architecture design. In this paper, we use software design patterns as a solution. This is due to their reusable software elements. Our principal objective is to propose other alternatives to the informal visual description of software architectures. In past work, we have studied Service Oriented Architectures. We used SOA design patterns with standard formal notations. This work is a continuation to the past one. We apply our approach on design patterns for the Internet of Things. We introduce a refinement-based approach for modeling IoT design patterns. It takes advantage of graphical modeling and formal method. It is organized around two main axes. The first axis is to provide modeling solutions in conformance with the UML standard language. The second axis covers the general specification of design pattern models with the Event-B method. As a result, we propose a design support tool for IoT architectures based on IoT design patterns. It allows modeling of correct-by-design software systems.

## Introduction

The Internet of Things (IoT) is a complex domain of application that allows objects to exist on the Internet. Creating systems in this domain is a challenge because it involves both software and hardware, sensing and actuating devices, a communication infrastructure, in addition to storage constraints. For this, a variety of IoT design patterns have been proposed in various categories to address variety of issues [7]. They propose solutions for common and recurring problems to architects and designers in the IoT domain. Most of these patterns are presented visually and informally, there is no formal semantics associated with them. Hence, their meanings may be imprecise. They can lead to their misunderstanding and misuse.

To remedy this problem, we propose an approach that allows to model and specify these patterns with a formal notation that allows to reuse them correctly. Our objective is to prove the relevance of these patterns. We illustrate our approach with different pattern examples. We propose a graphical modeling of these patterns in order to describe both their structural and behavioral features. Then, we propose a generic formal specification of these patterns using the formal Event-B method. Finally, we develop a graphical editor describing our approach using the Eclipse modeling platform.

The rest of this paper is organized as follows. Section 2 focuses on the structural modeling of IoT design patterns and Sect. 3 focuses on the behavioral modeling. In Sect. 4, we present an application to a case study of our approach. Section 5 describes how to formally specify IoT design patterns with the Event-B method. In Sect. 6, we present our tool, which implements the proposed approach. Section 7 discusses related work. Section 8 concludes and gives future work directions.

## Structural Patterns Modeling

We provide a modeling solution for describing IoT design patterns using a visual notation based on the graphical UML language in order to give readable models. We first describe a meta-model, then we present a model instance of the design pattern. The metamodel extends the component diagram of UML 2.0 (Unified Modeling Language). The use of UML is motivated by four distinct rationales: (i) It is a standard modeling language defined by OMG. (ii) It is used to describe software architectures. (iii) Component diagrams of UML allow us to represent structural features of patterns. (iv) Sequence diagrams of UML allow us to represent behavioral features of patterns.

Structural features of patterns are generally specified by the types of entities. The configuration of the entities is also described in terms of static relationships between them [16]. We model structural features of design patterns with the extended *Component* diagram. In the following, we present the proposed metamodel. An example of a corresponding model is presented and illustrated with case studies as follows.

### Metamodel

**Fig. 1.**
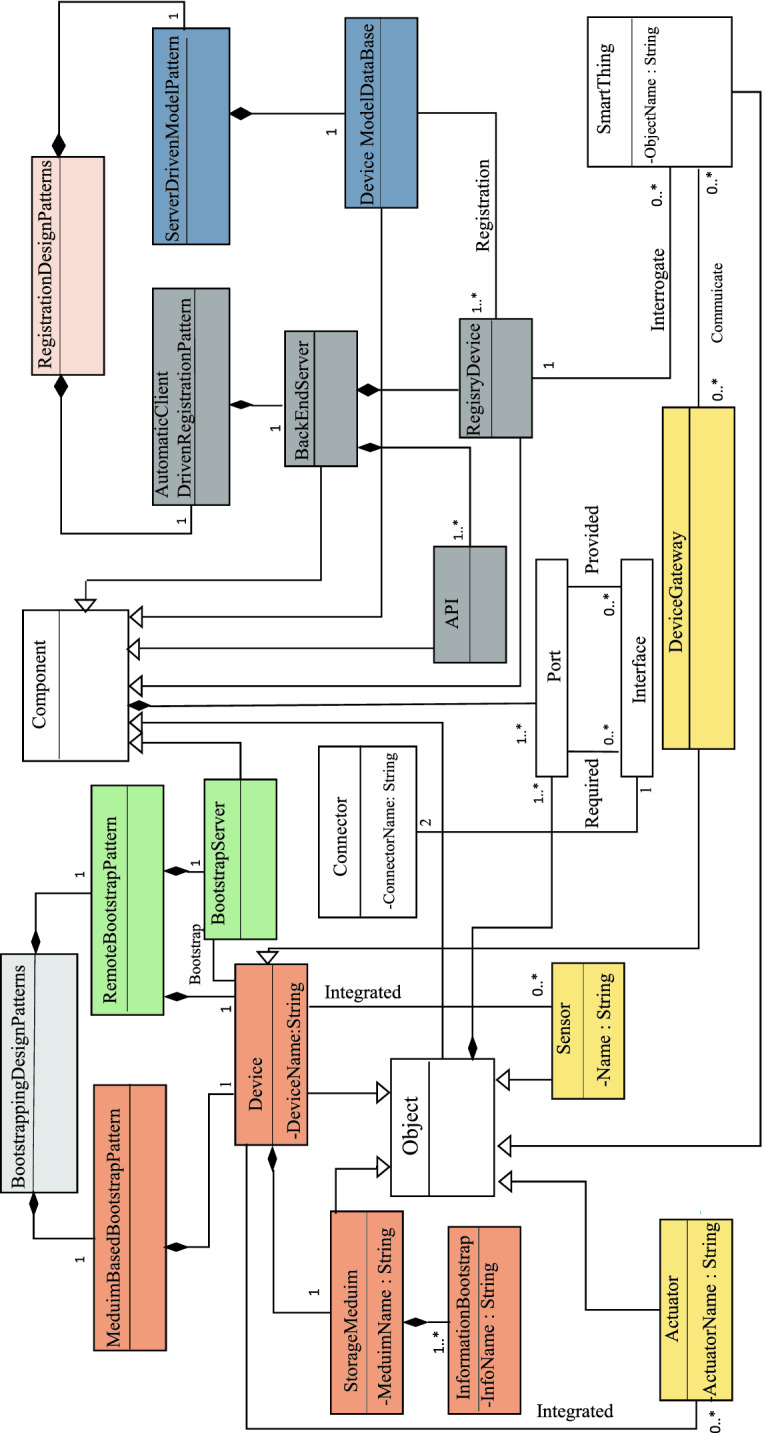
Metamodel of IoT design patterns

The extended *Component* diagram describes, by a set of concepts, the structure of an IoT architecture. We use it to describe the architecture of IoT design patterns. More specifically, it is to define the entities that can be involved in the pattern, their types and their dependencies (connections). The metamodel presented in Fig. 1 extends the metamodel of the component diagram of UML 2.0. In this metamodel, we concentrate on two categories of design patterns; “Bootstrapping Design Patterns” and “Registration Design Patterns”.

“Bootstrapping Design Patterns” allow configuring new devices. They are composed of “Medium Based Bootstrap Pattern” and “Remote Bootstrap Pattern”. “Medium Based Bootstrap Pattern” allows to configure a new device on-site through a removable storage medium inserted in the device. This support contains the necessary information for configuration. “Remote Bootstrap Pattern” is a configuration pattern used in case that a device is placed far away and is difficult to reach. The configuration in this case is done by downloading configuration information from a bootstrap server.

“Registration Design Patterns" allow to register the attributes and the features of a new device on the Back-end server. The registration is used to facilitate the communication and the interrogation with other connected objects. There are many registration patterns. In this work, we present two patterns. So “Registration Design Patterns" are composed of “Automatic Client Driven Registration Pattern” and “Server Driven Model Pattern”. The “Automatic Client Driven Registration Pattern” allows the device to register on the Back-end server via an API call. The “Server Driven Model Pattern” is used to create a device model that includes its description and functionality.

The basic elements of the metamodel are:  

**Component and Object:** Entities, that make up the architecture of an IoT design pattern, can be either *Components* or *Objects*. All objects are components, but not all components are necessarily objects. An object can be connected to the internet, it can receive and send data.
**Port:** Entities can have *Ports* that constitute interaction points with their environment. These *Ports* are related to one or more *provided* or *required* *Interfaces*.
**Interface:** The interfaces are the points of communication that allow interaction with the environment. For an entity, there are two types of interfaces. The *Provided Interfaces* describe the services provided by the component. The *Required Interfaces* describe the required services that other components must provide for the good functioning of component. These interfaces are specified via the ports.
**Connector:** The communication path between Entities within an architecture is called a *Connector*. It ensures the link between a *Provided* port and a *Required* port to form a complete and coherent system.
**Device:** A device is an Object. It is the entry point of the physical environment, it is used to process sensor data and to control actuators.

 

### General Pattern Model

In this section, we present two general pattern models as instances of the proposed metamodel. We have used different notations that can be used as a graphical description of the entities presented in the model. We are based on the work of Reinfurt et al. [8].

**Fig. 2.**
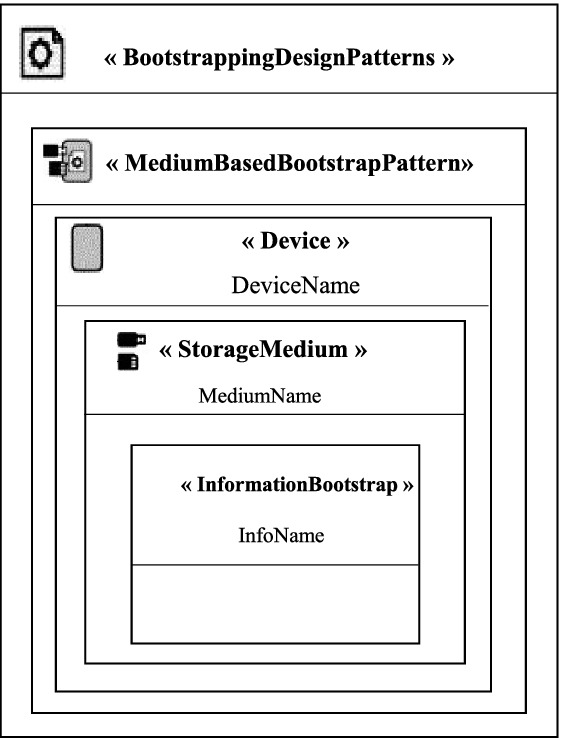
General pattern model of the Medium Based Bootstrap Pattern

**Fig. 3.**
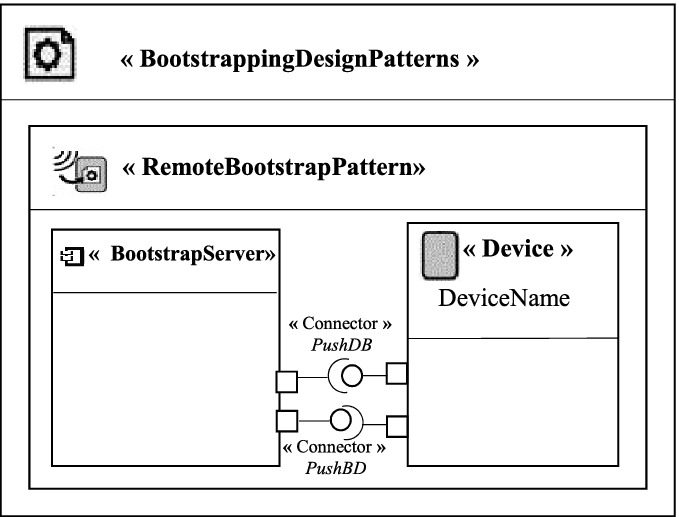
General pattern model of the Remote Bootstrap Pattern

There are two possible general models depending on the location of the device. If the device is placed locally, we use the “Medium Based Bootstrap Pattern” as a solution to configure the new device. If it is placed at a distance, we use the “Remote Bootstrap Pattern” as a solution. In Fig. 2, we represent the general pattern model of the “Medium Based Bootstrap Pattern”. The solution proposed by this pattern to configure a new local device is to use an object of type *Storage Medium* containing information configuration. In Fig. 3, we represent the general pattern model of the “Remote Bootstrap Pattern”. The solution proposed by this pattern to configure a new remote device is to use a component of type *BootstrapServer* allowing the upload of the configuration information using the PushBD connector.

**Fig. 4.**
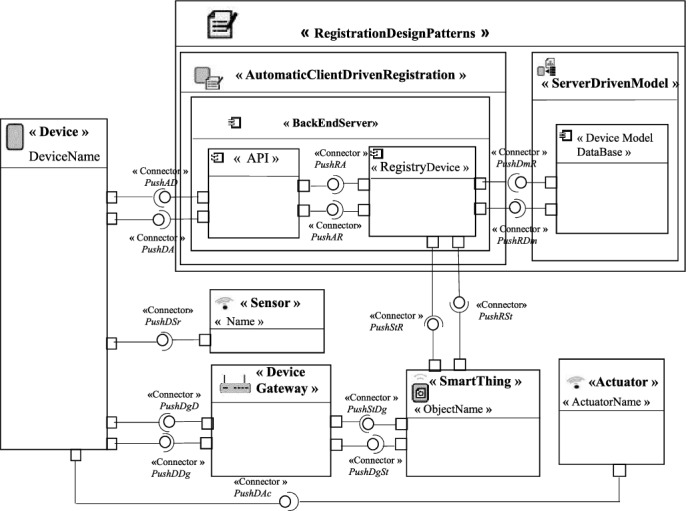
General pattern model of the Registration Design Pattern

In Fig. 4, we represent the general pattern model of the “Registration Design Pattern”. The solution proposed by this pattern to register a new device. The device is related to the *BackEndServer* with a connector named PushDA in order to be registered on it via an *API* call. Meta-data entered by the device are recorded in the *RegistryDevice* through the PushAR connector. The *RegistryDevice* component has a connector named PushRDm to store a device template in a database component named *Device Model DataBase* of the “Server Driven Model pattern”. A device can integrate an object of type *Sensor* or an *Actuator*. All objects of the patterns have ports to communicate with others.

## Behavioral Patterns Modeling

**Fig. 5.**
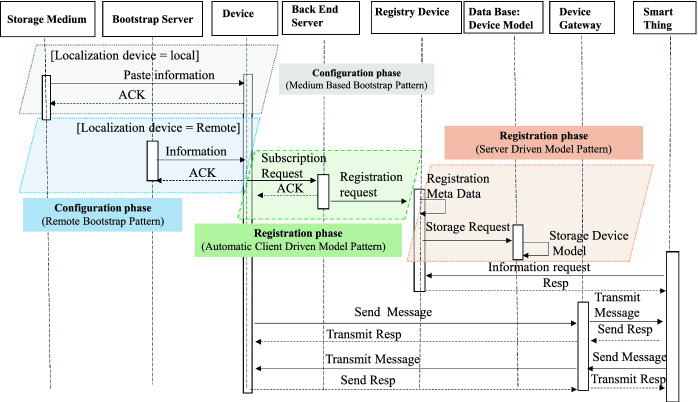
Sequence diagram of the used IoT patterns

To model behavioral features of the design patterns, we use the UML 2.3 sequence diagram. We describe through this diagram successive interactions between the different entities of the IoT application in order to represent the two categories of the design patterns. Figure 5 represent the sequence diagram that illustrates this behavior. We grouped the interactions into two phases.

- **Configuration phase:**
  - **Local configuration:** In the configuration phase and at the “Medium Based Bootstrap Pattern” level, the configuration is done by cutting the storage medium configuration information (*Storage Medium*) to the device.
  - **Remote configuration:** The configuration at the “Remote Bootstrap Pattern” is done by downloading information from a *Bootstrap Server* to the device.
- **Registration phase:** In the registration phase, the device triggers a registration process on the *Back-end Server*. After the registration, the metadata provided by the device are registered in the *RegistryDevice*. Subsequently, an instance of the model of this device is stored in a *Device Model Database*.

If the device, go through the configuration and the registration phases, it becomes able to create communication links with other connected objects. The exchange of messages between them is done through a communication intermediary (*Device Gateway*). A connected object (smart thing) can interact directly with the *RegistryDevice* to retrieve information about a device if it is offline.

## Case Study: Smart Home

To validate our approach, we chose to apply a case study in the IoT application domain called “Smart Home”.

A smart home is usually made up of remote-controlled automated components which can be doors, windows, lamps, etc. It can include several other components that can be monitored and controlled remotely. Most of these components can be controlled by a mobile device or a computer. In our case study, we add a new device (a camera) to a smart home. This device makes it possible to control the various rooms of the house. For example, if a door or a window left open, the camera informs the user immediately through a notification sent to their smartphone. It can also send alerts when it detects unknown faces.

First, the camera is added without any information to initiate its first connection. We then apply the Meduim Based Bootstrap design pattern to have its configuration information. This information is inserted into the device through a memory card. Second, we go through the registration procedure on the main server (BackEndServer). This procedure is done through the use of the two patterns of the Registration Design Patterns category which are the automatic client driven registration pattern and the server driven model pattern which allow registering the device on the main server. Finally, the camera became able to communicate and create connection links with its communication partners. The camera communicates with the user’s smartphone to notify him of what is happening in real-time.

**Fig. 6.**
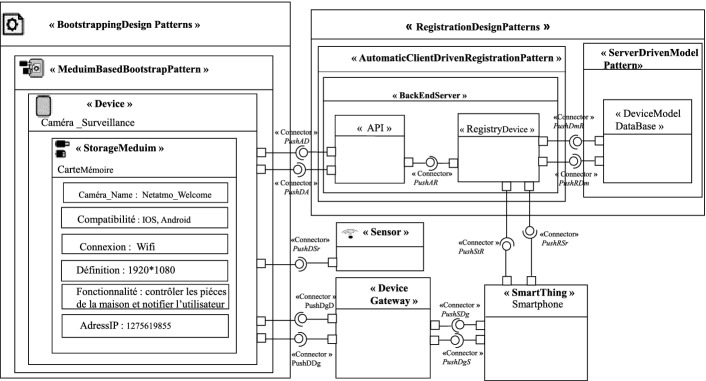
Smart home case study

We model this application through the use of the model shown in Fig. 6. The camera is associated with an object of type “Device”, the memory card is defined as an object of type “Storage Medium” and the Smart phone is associated as an object of type “SmartThing”. The propagation of events between objects is done through a *DeviceGateway*.

## Patterns Specification

UML, as semi-formal language offers several benefits to the definition of IoT design patterns, such as visual and standard notation. This graphical aspect is certainly interesting and useful to an architect, in the sense that graphic design is easy. However, the fact that UML lack a precise semantics is a serious drawback because this language did not allow checks which we must carry. So, pattern models generated at the modeling approach can be ambiguous and imprecise. In addition, during the modeling phase, the architect can easily fall into the error. This is due to the absence of a precise formal semantics of UML that do not provide rigorous tools for verification and proof. However, any error or any bad modeling of a design pattern can cause serious problems that generate bad consequences.

Thus, ensuring the reliability and the correctness of IoT design patterns is a goal that we have fixed. For this, we propose an approach to formally specify design patterns by using the formal method Event-B that is well suited to our needs and goals. Thus, each diagram graphically modeled will be accompanied by a formal semantics. This approach allows the validation of the modeling part and ensure the verification of the relevant properties of design patterns.

Event-B method is well-suited for specifying IoT design patterns: (i) The primary concept in doing formal developments in Event-B is that of a model. It is made of several components of two kinds: machines and contexts. Machines contain the dynamic parts of a model, whereas contexts contain the static parts of a model [1]. Thanks to this classification, Event-B allows the specification of structural and behavioral features of design patterns. (ii) Refinement techniques proposed by this method allow us to build patterns gradually and at different abstraction levels. (iii) Mathematical proofs allow verifying model consistency and consistency between refinement levels. (iv) The most important reason to use Event-B method is the availability of a supporting tool called the Rodin platform [2]. It is an Eclipse-based tool set that provides effective support for modeling and automated proof. The platform is open source and is further extendable with plug-ins. A range of plug-ins have already been developed including ones that support animation and model checking like the Prob plug-in [5] that we used.

Extended *Component* diagram that model structural features of design patterns are transformed to a context in the Event-B method in which we specify entities of the architecture and their relations. The *Sequence* diagram is transformed into a machine in Event-B in which we specify events made between entities of the patterns. This transformation is proposed in order to attribute formal notations to IoT design patterns for the purpose of checking their design correctness in a second step. We explicitly defined a refinement strategy to follow. This strategy is interesting because it defines the pattern development process and improves the quality of the obtained models, and therefore the success of the formal development process. We defined specification levels by using a step-wise development approach.

## Tool Support

**Fig. 7.**
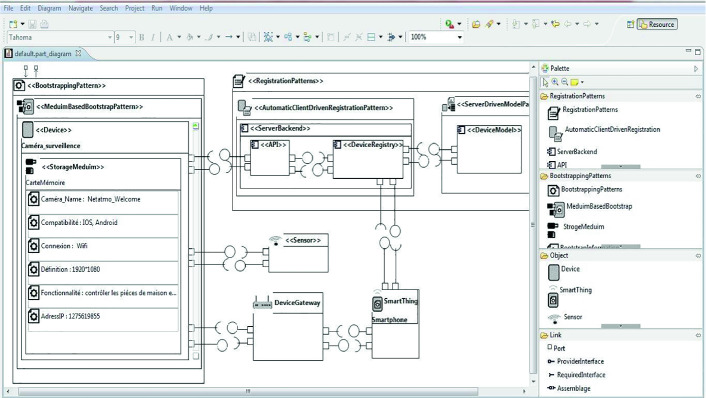
The tool editor

We developed a graphical modeling tool that implements our approach; it ensures an easy and efficient modeling way for users. With our tool, we aim to make concrete the aforementioned concepts. The architect can model the solution of the IoT design patterns using an Eclipse plug-in that we propose. The tool, in its development, is based on EMF^1^ (*Eclipse* *Modeling* *Framework*) [10]. This was chosen since we use models, which are basic building units, to develop our approach (Fig. 7).

## Related Work

Research connected to design patterns in the field of software architecture, are mainly classified into four branches of work according to their architectural style. The first is about design patterns for Object-Oriented Architectures, the second is about design patterns for Enterprise Application Integration (EAI), the third is for Service Oriented Architectures (SOA) and the fourth one is for connected object architectures.

Most of the proposed design patterns are described with a combination of a text description and a graphical representation sometimes using a proprietary notation in the aim of making them easy to understand. However, these descriptions make patterns ambiguous and may lack details. Some work so have proposed the semi-formal representations of these patterns using modeling languages [4]. Some other works use or provide formal languages based on mathematical notation for a precise pattern specification [16]. However, these approaches require knowledge of mathematics and first order logic to use them. Some research has chosen to combine the semi-formal and formal representations of patterns. This representation ensures a better understanding and precision of patterns. Generally speaking, there is a consensus on the elements that make up and define a design pattern. However, there is no consensus on the specification of the patterns.

In past work [11, 13] we focused on both the modeling, the formal specification and the composition of SOA design patterns [12, 14] and established the link between them with an automatic transformation [15]. We used the SoaML language for the pattern modeling that ease the understanding of pattern models. For the pattern specification, we used the Event-B formal method in order to attribute formal notations to SOA design patterns for the purpose of checking their design correctness.

In this work, we are interested with the IoT design patterns. In this context we find several researchers who proposed a set of IoT design patterns in various categories. Eloranta et al. [3] proposed patterns for the construction of distributed control systems. Qanbari et al. [6] presented four patterns for the supply, deployment, orchestration and monitoring shipboard applications. Reinfurt et al. [7, 9] have published patterns for device power supply, operation and communication modes and a number of IoT design models. All these patterns are described with a visual and informal notation. There is no formal semantics associated. There is no research work that deals with the modeling of IoT patterns. In this paper, we present the modeling of IoT design patterns proposed by Reinfurt et al. [7].

## Conclusions

In this paper, we presented an approach that allows to model and specify connected object architecture design patterns. In particular modeling the “Bootstrapping Design Patterns” category and the “Registration Design Patterns” category. The modeling phase consists of presenting models of the design patterns in order to present a meta-model that presents an abstract view of a model of the patterns. Subsequently, we described the structural and behavioral features of the pattern. Then, we formally specified these design patterns using the formal Event-B method. Finally, we developed a plug-in under the Eclipse Modeling platform that offers a graphical editor for modeling IoT design patterns. Currently, the transition from the SoaML modeling to the formal specification is achieved manually, we are working on automating this phase by implementing transformation rules.

## Footnotes

1 https://wiki.eclipse.org/Eclipse_Modeling_Framework.
